# Plexiform Neurofibroma: A Rare Tumor of Submandibular Salivary Gland

**DOI:** 10.4103/2006-8808.73622

**Published:** 2010

**Authors:** T. Y. Shekar, Gautam Gole, Shailaja Prabhala, Sheetal Gole

**Affiliations:** *Department of General Surgery, India*; 1 *Department of Pathology, Kamineni Institute of Medical Sciences, Sreepuram, Narketpally, Dist - Nalgonda, Andhra Pradesh, India*

**Keywords:** Plexiform neurofibroma, sialadenitis, sialolithiasis, submandibular salivary gland

## Abstract

A 15-year-old boy presented with swelling in the submandibular region. X-ray of the swollen part showed faint radio opaque shadow. A provisional diagnosis of sialadenitis with sialolithiasis was made. Excised mass was reported histopathologically as plexiform neurofibroma of submandibular salivary gland.

Plexiform neurofibroma of the salivary gland is a rare benign tumor often present in the parotid gland. It is very rare in submandibular salivary gland. It is a slow growing, locally infiltrating tumor.

## INTRODUCTION

Neurofibromas constitute only 0.4% of all salivary neoplasms. Plexiform neurofibromas of the salivary glands are rare, often presenting in the parotid gland. They are very rare in submandibular salivary gland.[1,2] They are slow growing, locally infiltrating tumors. Only five cases are reported so far in the literature.[3] Probably this is the sixth one. We are presenting this case because of its rarity of presentation.

## CASE REPORT

A 15-year-old boy was admitted with a swelling in the submandibular region, which rapidly progressed in size. There was no history of pain or increase in size of swelling while taking meal. On examination, a firm swelling in the submandibular region of size 8 × 5 cm, with ovoid shape and well-defined borders was present. It was bidigitally palpable. No café-au-lait spots were present over the body. A faint radio-opaque shadow, suggestive of calculus was noticed in the region of the gland. Provisional diagnosis was chronic sialadenitis with sialolithiasis. Fine needle aspiration cytology (FNAC) was reported as chronic sialadenitis.

**Figure 1:**
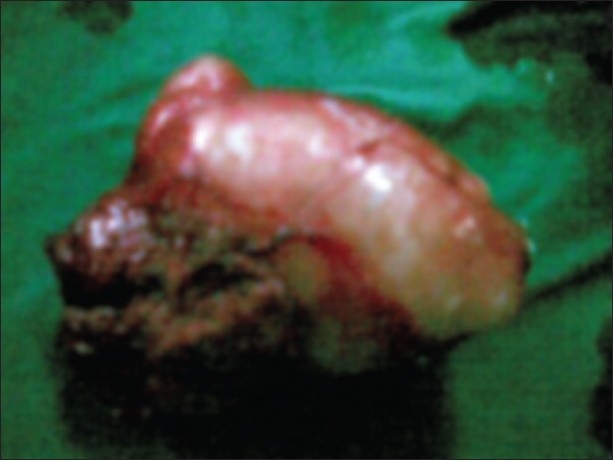
Specimen of plexiform neurofibroma of submandibular gland

Intraoperatively, mass was arising from submandibular salivary gland. A tubular growth was extending from the mass into the adjacent tissue [Figure 1].

**Figure 2:**
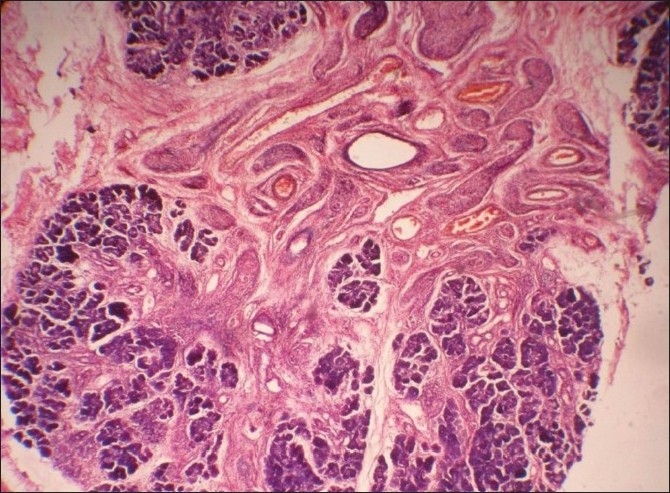
H and E section (×10) shows salivary gland tissue with interspersed, thickened nerve bundles

**Figure 3:**
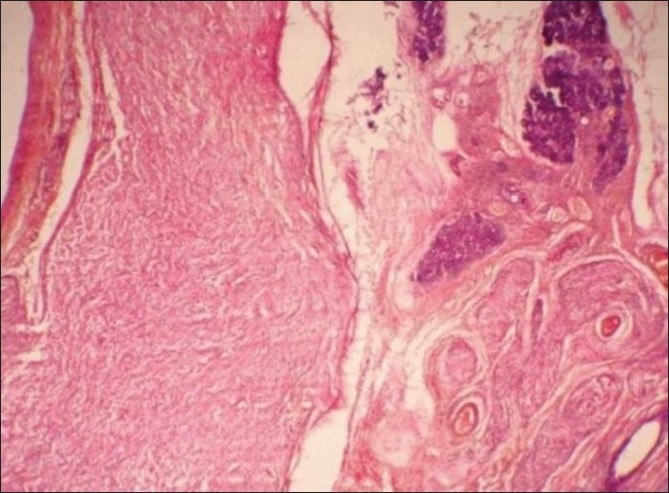
H and E section (×10) shows markedly enlarged nerve segment and thickened nerve bundles within the salivary gland

Gross specimen was gelatinous and vaguely nodular, showing greyish white to grey yellow areas. No calculus was found in the specimen. Microscopic picture showed cellular and nodular area of tumor tissue. Central nerve fiber bundles surrounded by neurofibroma tissue were seen in an abundant mucoid matrix and collagen background. Adjacent salivary gland also showed thickened nodular nerve bundles between salivary lobules. No atypia was present. Features were suggestive of plexiform neurofibroma of the submandibular salivary gland [Figures 2 and 3].

## DISCUSSION

Neurofibromas are benign nerve sheath tumors which present in the following three forms: local discrete, generalized neurofibromatosis, and plexiform neurofibromas. Common sites of occurrence of plexiform neurofibroma are fifth cranial nerve and extremities. Neurofibromas constitute only 0.4% of all salivary neoplasms. Plexiform neurofibromas of the salivary glands are rare, usually presenting in the parotid gland. They are very rare in submandibular salivary gland.

Plexiform neurofibromas are diffuse enlargements of multiple fascicles of the nerves and its branches, leading to thickening of nerves. They grow along nerves extending into the surrounding tissue. They are slow growing and locally infiltrating benign tumors. When located deeply, they may have greater chance of malignancy. Large plexiform neurofibromas of submandibular salivary gland may be associated with pressure symptoms on trachea and pharynx. Sometimes they may extend into spinal canal and compress the spinal cord. They grow rapidly in children, during puberty, and in pregnancy.

Plexiform neurofibromas are frequently seen in patients with Type I neurofibromatosis and undergo malignant changes in 2% of the cases. Patient with plexiform neurofibroma without family history or without features of Type I neurofibromatosis require genetic work up. In such cases, tumor may present as a result of local somatic mutation and patient may not transmit the disease to their offspring.[4]

Neurofibromas have been reported in parotid glands.[5,6] Of 300 neurofibromas in the minor salivary gland tumors, two were found to be plexiform neurofibromas.[7] Plexiform neurofibromas are exceedingly rare in submandibular and sublingual salivary glands.[1,2,8]

A search in the literature revealed only five cases of plexiform neurofibromas of submandibular salivary gland [Table 1].

**Table 1:** Comparative data of five reported cases of plexiform neurofibroma

Author	Year	Reference no.	Age of patient (years)	Sex	Presenting symptom	Surgery	Pathological type
Bourgeois J.M., Radhi J, DenEi L.	2001	[1]	3	Male	Submandibular mass with multiple neurofibromatosis	Excision of submandibular mass	Plexiform neurofibroma
Weitzner S.	1980	[2]	3	Female	Submandibular mass with multiple neurofibromatosis	Excision of submandibular mass	Plexiform neurofibroma
Tsutsumi T. Komat Suzaki. A	1996	[5]	29	Female	Submandibular mass	Excision of submandibular mass	Plexiform neurofibroma
Derekoy S, Sefali M	2000	[9]	21	Male	Submandibular mass with multiple neurofibromatosis	Excision of submandibular mass	Plexiform neurofibroma
Aribandi M, Wood W.E., Elston D.M. and Weiss D.L	2006	[3]	6	Female	Submandibular mass with multiple neurofibromatosis	Excision of submandibular mass	Plexiform neurofibroma

Of five cases, three were females and four of the above five cases presented with Type I neurofibromatosis. However in our case, there was no Type I stigma present.

If we consider FNAC to diagnose plexiform neurofibroma, its diagnostic yield appears to be low. A plexiform neurofibroma may be suspected in the appropriate setting with the help of cross sectional imaging. Computed tomography (CT) imaging may show conglomerate multilobulated masses that may appear as a ’bag of worms.’ There is hypoattenuation with heterogenous contrast enhancement. A characteristic ’branching’ hypoattenuated mass on CT, with branching tubular masses extending into adjacent tissue, could virtually be diagnostic.[10] In our case CT was not done.

Histopathologically, there is diffuse cylindrical enlargement of multiple fascicles of the nerve and its branches. There is a myxoid matrix containing Schwann cells, nerve fibers, mast cells and perineurial, endoneurial fibroblasts.

Surgical excision is the treatment of choice. Recurrence may occur. It is reported in 20% of the patients with plexiform neurofibroma after complete resection and increases to 44% with incomplete resection (where there is involvement of vital structures).[3]

## CONCLUSION

Plexiform neurofibroma is an extremely rare tumor. It is a slow growing tumor in the submandibular region. It is more common in children. CT is somewhat diagnostic. FNAC may be helpful. Final diagnosis can be achieved by excision biopsy. Frequently, patient desires excision for cosmetic reason. Recurrence is common in case of incomplete removal.

## References

[1] Bourgeois JM, Radhi J, DenEi L (2001). Plexiform neurofibromas of the submandibular gland in a child. Can J Gastroenterol.

[2] Weitzner S (1980). Plexiform neurofibromas of the submandibular gland in children. Oral Surg Oral Med Oral Pathol.

[3] Aribandi M, Wood WE, Elston DM, Weiss DL (2006). CT Features of plexiform neurofibroma of the submandibular gland. AJNR Am J Neuroradiol.

[4] Scheithaur BW, Woodruff JM, Erlandson RA, Rosai J (1999). Tumours of the Peripheral Nervous System. Atlas of tumor pathology. Fascicle 24, 3^rd^ series.

[5] Tsutsumi T, Suzaki KA (1996). Solitary plexiform neurofibromas of the submandibular gland. J Laryngol Otol.

[6] Seifert G, Meihike A, Haubrich J (1986). Pathology, diagnosis, treatment, facial nerve surgery.

[7] Castro EB, Huvos AG, Strong EW (1972). Tumours of the major salivary glands in children. Cancer.

[8] Kahwaji G, Hamdan AL, Mufarij A (2000). Plexiform neurofibromas of the submandibular gland. Otolaryngol Head Neck Surg.

[9] Derekoy S, Sefali M (2000). Plexiform neurofibromas of the submandibular gland. J Laryngol Otol.

[10] Lin J, Martel W (2001). Cross sectional image of peripheral nerve sheath tumors: Characteristic sign on CT, MR imaging and sonography. AJR Am J Roentgenol.

